# The missing link between standing- and traveling-wave resonators

**DOI:** 10.1515/nanoph-2022-0304

**Published:** 2022-08-19

**Authors:** Qi Zhong, Haoqi Zhao, Liang Feng, Kurt Busch, Şahin K. Özdemir, Ramy El-Ganainy

**Affiliations:** Department of Physics, Michigan Technological University, Houghton, MI 49931, USA; Department of Electrical and Computer Engineering, Michigan Technological University, Houghton, MI 49931, USA; Department of Electrical and Systems Engineering, University of Pennsylvania, Philadelphia, PA 19104, USA; Department of Materials Science and Engineering, University of Pennsylvania, Philadelphia, PA 19104, USA; Department of Engineering Science and Mechanics, The Pennsylvania State University, University Park, PA 16802, USA; Materials Research Institute, The Pennsylvania State University, University Park, PA 16802, USA; Humboldt-Universität zu Berlin, Institut für Physik, AG Theoretische Optik & Photonik, D-12489 Berlin, Germany; Max-Born-Institut, Max-Born-Straße 2A, 12489 Berlin, Germany

**Keywords:** integrated photonics, optical resonators, sensing

## Abstract

Optical resonators are structures that utilize wave interference and feedback to confine light in all three dimensions. Depending on the feedback mechanism, resonators can support either standing- or traveling-wave modes. Over the years, the distinction between these two different types of modes has become so prevalent that nowadays it is one of the main characteristics for classifying optical resonators. Here, we show that an intermediate link between these two rather different groups exists. In particular, we introduce a new class of photonic resonators that supports a hybrid optical mode, i.e. at one location along the resonator the electromagnetic fields associated with the mode feature a purely standing-wave pattern, while at a different location, the fields of the same mode represent a pure traveling wave. The proposed concept is general and can be implemented using chip-scale photonics as well as free-space optics. Moreover, it can be extended to other wave phenomena such as microwaves and acoustics.

## 1 Introduction

Light is a very peculiar form of energy that constantly travels from one point to another, which makes it difficult to store or “freeze” it in one place. This, however, can be effectively overcome by using optical resonators that utilize feedback mechanisms together with wave interference effects to recycle light along periodic trajectories. Depending on the resonator’s geometry, these trajectories may intercept each other in opposite directions forming standing-wave patterns with a vanishing Poynting vector. Alternatively, they may form closed loops that support degenerate circulating traveling-wave modes (clockwise (CW) or counterclockwise (CCW)) with a non-vanishing Poynting vector along the loop direction. This ability to confine and trap light has enabled several scientific breakthroughs over the past few decades. Importantly, recent technological progress in micro- and nano-fabrication has enabled the realization of on-chip optical resonators with spatial dimensions comparable to the wavelength of the trapped light, or even smaller [1] with a wide range of applications including microlasers [2–8] and sensing [9–14], just to mention few examples. Despite the large variety in their designs (microrings, microdisks, photonic crystals, Bragg structures, etc.), sizes, and material systems, optical resonators are typically classified into one of the aforementioned categories, i.e. standing- or traveling-wave devices [15–17]. This classification is generally accepted as complete. Thus, research in the field of optical resonators has focused on applications of these resonators and implementations of novel designs with unique features. In particular, standing-wave resonators can be engineered to support small mode volumes and high quality factors, which makes them perfect choice for engineering quantum light–matter interactions [18–26]. On the other hand, traveling-wave resonators are the preferred platform for (classical and quantum) nonlinear optics due to the ability to engineer the interaction between different wave components and the unidirectional propagation properties of these modes which facilitates the input and output coupling [27, 28]. In addition, it was shown previously that the interaction between an atom and a standing-wave pattern of light depends on whether the latter is generated in a standing-wave resonator or as a result of interference between counter-propagating waves in a traveling-wave resonator [29].

In this work, we show that this classification scheme for optical resonators (as standing- or traveling-wave resonators) is not complete. Instead, we reveal a new type of optical modes supported by certain resonator structures that represents a missing link between these two categories. Specifically, we propose a new resonator concept that supports an optical mode exhibiting hybrid standing- and traveling-wave patterns simultaneously.

This article is organized as follows. First, we introduce a general concept outlining the behavior of the proposed resonator without a reference to a particular structure. Afterwards, we discuss an implementation based on standard chip-scale photonics technology. To gain insight into the modal structure of the proposed resonator, we present a detailed analysis of its modal features using a scattering matrix approach. Next, we confirm our results by using full-wave finite element simulations. Finally, we investigate the effect of local perturbation due to a small scatterer on the eigenmodes of the proposed hybrid-wave resonator.

## 2 General concept

**Figure 1:**
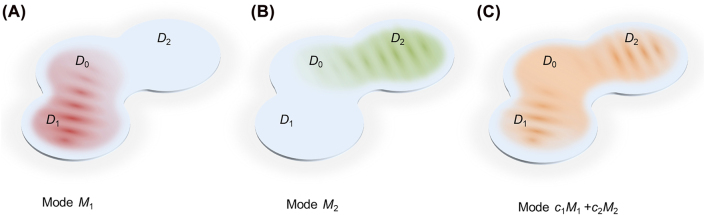
The concept of a hybrid-wave resonator. A resonator structure that supports two degenerate standing-wave modes such that (A) mode *M*_1_ resides in domain *D*_0_ ∪ *D*_1_, and (B) mode *M*_2_ resides in domain *D*_0_ ∪ *D*_2_. (C) It is possible that a proper linear superposition between *M*_1_ and *M*_2_ can result in new modes that preserve the standing-wave character in domains *D*_1,2_ while at the same time form a traveling wave in domain *D*_0_. In the figure, standing and traveling waves are schematically represented by interference fringes and uniform field distributions respectively.

We start by considering a generic concept of optical resonators that supports modes overlapping only partially with the physical structure of the resonator. For simplicity, assume that such a resonator can be divided into three domains (extension to more domains is straightforward): *D*_0_, *D*_1_ and *D*_2_ (the exact definition of the domain boundaries is not important). Furthermore, assume that it supports two degenerate standing-wave modes *M*_1,2_ such that the field distribution of *M*_1_ mainly resides in *D*_0_ ∪ *D*_1_, and similarly the field associated with mode *M*_2_ resides in *D*_0_ ∪ *D*_2_ as shown schematically in Figure 1A and B, respectively. The continuity conditions for the electromagnetic field across domain boundaries dictate that the field distributions associated with modes *M*_1,2_ must be different across domain *D*_0_. Let us now consider a general superposition of these two modes. It may be anticipated that, a new set of basis (owing to the degeneracy, there are infinitely many bases) can be constructed such that the standing-wave nature of modes *M*_1,2_ in domains *D*_1,2_ remain unaltered and yet the field distribution in *D*_0_ forms a traveling-wave pattern due to a particular linear superposition of modes *M*_1,2_ (Figure 1C), akin to the relation cos(*kz*) + *i* sin(*kz*) = exp(*i* *kz*). Such a “mutant” resonator, if it exists, will support a “mutant” optical mode that, in some properly chosen basis, exhibits purely standing- and traveling-wave patterns at the same time—a feature that, to the best of our knowledge, has never been discussed before. We will refer to such a resonator as a hybrid-wave resonator. So far, we have kept the discussion abstract. In what follows, we show that this abstract concept can be implemented in realistic optical resonator designs. In the main text, we focus on chip-scale devices but we note that it is straightforward to extend the discussion to implementations using free-space optics.

## 3 Integrated photonics implementation

**Figure 2:**
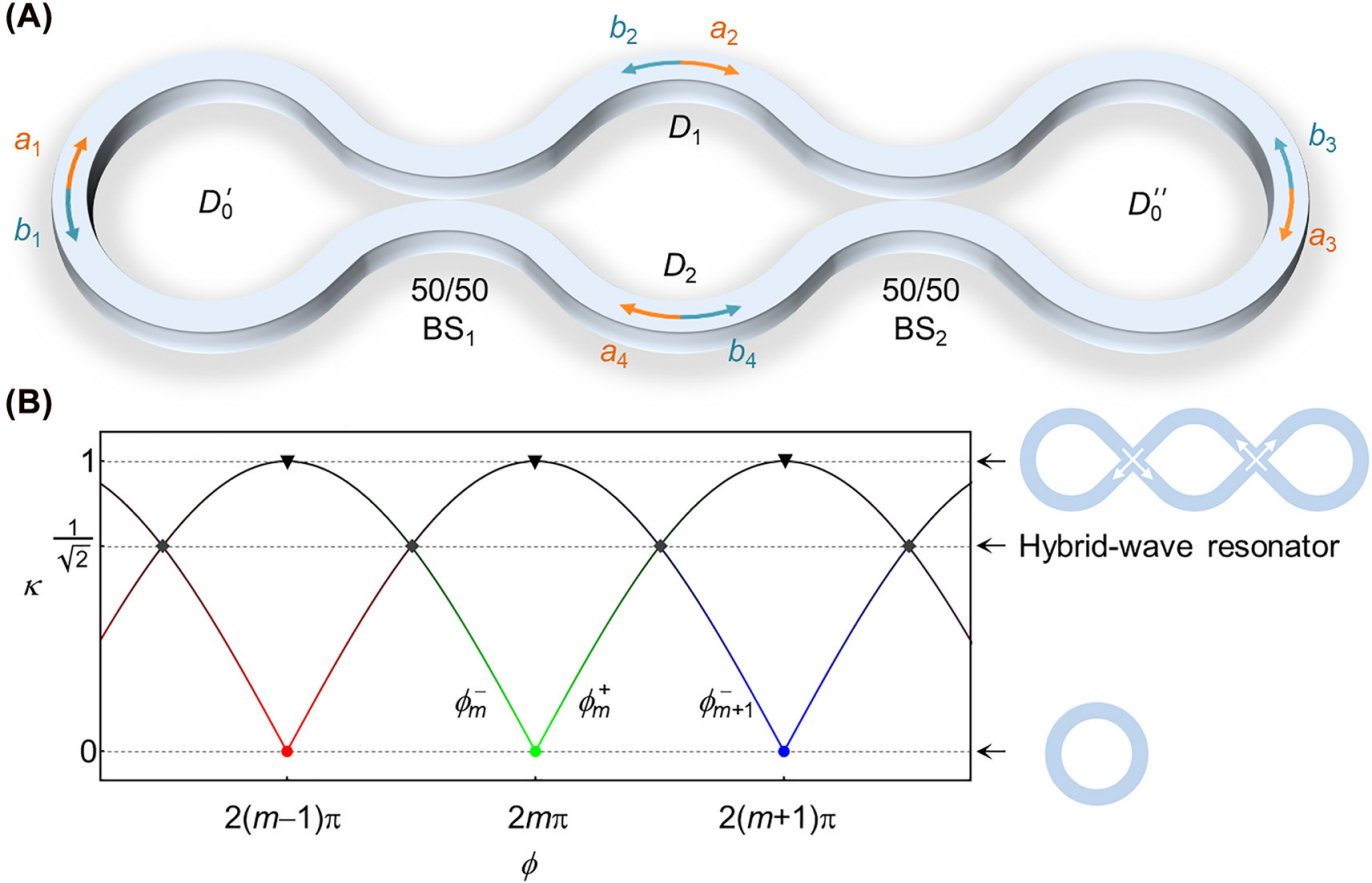
An implementation of a hybrid-wave resonator in integrated photonic platforms. (A) A hybrid-wave resonator can be constructed by deforming a ring resonator to introduce two 50/50 beam splitters, BS_1,2_. The resonator can be divided into three domains: $D_{0}=D_{0}^{\prime }\cup D_{0}^{\prime \prime }$ is the union of the left and right side rings, and domains *D*_1_ and *D*_2_ represent the top and bottom middle sections, respectively. The field amplitudes at each location and their traveling directions are indicated on the figure and labeled as *a*_*i*_ and *b*_*i*_, *i* = 1, 2, 3, 4. (B) Resonant frequencies (horizontal axis) as a function of the beam splitter coupling coefficient *κ* (vertical axis). Note that each *κ* value represents an independent resonator structure. Horizontal dashed lines indicate values of *κ* for which the spectrum is doubly degenerate. The limit *κ* = 0 corresponds to conventional microring resonator with two degenerate traveling-wave modes in clockwise and counterclockwise direction while the limit *κ* = 1 corresponds to degenerate “knotted” modes. Hybrid-wave modes exist for $\kappa =1/\sqrt{2}$ as discussed in detail in the main text.

To demonstrate that the concept discussed above can be realized by standard optical components, here we consider an implementation based on integrated photonics as shown in Figure 2A (possible realizations based on free-space optics are discussed in Supplementary Materials A). The structure consists of three open ring sections connected by two beam splitters (labeled as BS_1,2_). The outer rings here act as Sagnac loop reflectors [30]. We note that even though variants of this geometry have been considered before for building various optical devices for different applications such as sensing, lasing, and information processing [31–35], the peculiar feature that we highlight in this work has escaped attention.

To investigate the modal structure of this resonator, we will employ a scattering matrix analysis along the junctions indicated in Figure 2A. Away from the beam splitter junctions, the field amplitudes can be decomposed into two traveling waves in opposite directions as shown in Figure 2A. Within the context of scattering matrix formalism [17, 36, 37], the relations between these amplitudes are given by:

(1) $$\begin{array}{cc} {\left[a_{1},b_{1}\right]}^{\text{T}} & =S_{c}{\left[a_{4},b_{2}\right]}^{\text{T}},\\ {\left[a_{4},b_{2}\right]}^{\text{T}} & =S_{c}{\left[a_{3},b_{3}\right]}^{\text{T}},\\ {\left[a_{3},b_{3}\right]}^{\text{T}} & =S_{c}{\left[a_{2},b_{4}\right]}^{\text{T}},\\ {\left[a_{2},b_{4}\right]}^{\text{T}} & =S_{c}{\left[a_{1},b_{1}\right]}^{\text{T}}, \end{array}$$

where *S*_*c*_ = exp(*i* *ϕ*/4)*S*_*b*_ and $S_{b}=\left[\begin{array}{cc} \tau & i\kappa \\ i\kappa & \tau \end{array}\right]$ is the scattering matrix of each beam splitter. Here, *τ* and *κ* are the field transmission and coupling coefficient of each beam splitter, and in the absence of any loss, they satisfy *τ* ^2^ + *κ* ^2^ = 1. For the special case of a 50/50 beam splitter, which is relevant to our discussion later, $\tau =\kappa =1/\sqrt{2}$ . The phase term *ϕ* is defined as *ϕ* = 2*πn*_eff_ *Lf*/*c*, where *n*_eff_ is the effective guiding index, *L* is total length across the perimeter of the resonator, *f* is the frequency and *c* is the speed of light in vacuum. The numerical factor 1/4 in the expression of *S*_*c*_ arises because each wave component travels one quarter the length structure between any two consecutive junctions. In the absence of dispersion, *ϕ* is a linear function of *f* and thus can be used directly to determine the resonant frequencies (the effect of dispersion is considered later in the full-wave simulations).

By successive substitution of the right side of each line in Eq. (1) from the expression in the next line, we find:

(2) $${\left[a_{1},b_{1}\right]}^{\text{T}}=S_{c}^{4}{\left[a_{1},b_{1}\right]}^{\text{T}}=e^{i\phi }S_{b}^{4}{\left[a_{1},b_{1}\right]}^{\text{T}}.$$

The resonant modes can then be obtained by imposing a consistency condition requiring the operator $\exp\left(i\phi \right)S_{b}^{4}$ to have an eigenvalue equal to unity [38]. In order to find the values of *ϕ* that satisfy the consistency condition and hence obtain the eigenfrequencies, we first note that the eigenvalues of *S*_*b*_ are given by *λ*_1,2_ = exp(±*i* *θ*) and the corresponding eigenvectors are **v**_1,2_ = [1,±1]^T^. Here, *θ* = arcsin(*κ*) ∈ [0, *π*/2] since *τ* and *κ* take only positive values. The consistency condition then reduces to exp(*i* *ϕ*) exp(±*i*4*θ*) = 1, which has two solutions given by $\phi_{m}^{\pm }=\pm 4\theta +2m\pi$ , where *m* is an integer. In order to better understand this result, we recall that in the absence of beam splitters (*κ* = 0) the eigenfrequencies are doubly degenerate (for each resonant frequency there are two modes, one propagating in the CW and the other in the CCW direction) and are given by *ϕ*_*m*_ = 2*mπ*. Introducing the beam splitters results in coupling between CW and CCW modes and thus lifts the degeneracy. As a result, each degenerate pair described by *ϕ*_*m*_ splits into two modes: blue-shifted $\phi_{m}^{+}$ and red-shifted $\phi_{m}^{-}$ . Interestingly, for identical 50/50 beam splitters, i.e. when $\kappa =1/\sqrt{2}$ corresponding to *θ* = *π*/4, the eigenmodes associated with $S_{b}^{4}$ become degenerate (this is not the case for the eigenvectors of *S*_*b*_) and hence the eigenmodes of the resonators form degenerate pairs satisfying the resonant conditions $\phi_{m}^{+}=\phi_{m+1}^{-}=\left(2m+1\right)\pi \equiv \phi_{m}$ , as shown in Figure 2B. Before we proceed, we emphasize that the above-predicted degeneracy is not a result of a particular geometric symmetry. For instance, the length of any of the curved sections in the four domains ( $D_{0}^{\prime }$ , $D_{0}^{\prime \prime }$ , *D*_1_ and *D*_2_) can be increased by a multiple of the operation wavelength without affecting the degeneracy despite the fact that it will break part of the geometric symmetries of the structure.

We now investigate the eigenmode structure associated with these newly formed degenerate modes. In principle, these eigenmodes can be expressed in any basis of the eigenvectors of $S_{b}^{4}$ . Choosing a particular basis fixes the vector ${\left[a_{1},b_{1}\right]}^{\text{T}}$ which can be then used to obtain all other field components through Eq. (1). Table 1 lists the field values for three different bases given by: (1) *B*_1_ = {**v**_1,2_}, **v**_1,2_ = [1,±1]^T^; (2) *B*_2_ = {**v**_3,4_}, **v**_3,4_ = [1,∓*i*]^T^; and (3) *B*_3_ = {**v**_5,6_}, **v**_5_ = [2,0]^T^, **v**_6_ = [0,2]^T^. These bases are related via the linear transformations: $v_{3,4}=\left[\left(1\mp i\right)v_{1}+\left(1\pm i\right)v_{2}\right]/2$ and **v**_5,6_ = **v**_1_ ± **v**_2_. Expressed differently, **v**_5,6_ can be also written as **v**_5_ = **v**_3_ + **v**_4_ and **v**_6_ = *i*(**v**_3_ − **v**_4_).

**Table 1:** Field components associated with the degenerate eigenmodes of the structure shown in Figure 2 as expressed in the three different bases *B*_1,2,3_.

Domains		$D_{0}^{\prime }$		*D*_1_		$D_{0}^{\prime \prime }$		*D*_2_	
Field components		*a*_1_	*b*_1_	*a*_2_	*b*_2_	*a*_3_	*b*_3_	*a*_4_	*b*_4_
*B*_1_	$M_{1}^{\left(1\right)}$	1	1	$e^{i\frac{\phi_{m}+\pi }{4}}$	$ie^{i\frac{3\phi_{m}+\pi }{4}}$	$ie^{i\frac{\phi_{m}}{2}}$	$ie^{i\frac{\phi_{m}}{2}}$	$ie^{i\frac{3\phi_{m}+\pi }{4}}$	$e^{i\frac{\phi_{m}+\pi }{4}}$
	$M_{2}^{\left(1\right)}$	1	−1	$e^{i\frac{\phi_{m}-\pi }{4}}$	$e^{i\frac{3\phi_{m}+\pi }{4}}$	$-ie^{i\frac{\phi_{m}}{2}}$	$ie^{i\frac{\phi_{m}}{2}}$	$-e^{i\frac{3\phi_{m}+\pi }{4}}$	$-e^{i\frac{\phi_{m}-\pi }{4}}$
*B*_2_	$M_{1}^{\left(2\right)}$	1	−*i*	$\sqrt{2}e^{i\frac{\phi_{m}}{4}}$	$i\sqrt{2}e^{i\frac{3\phi_{m}}{4}}$	$e^{i\frac{\phi_{m}}{2}}$	$ie^{i\frac{\phi_{m}}{2}}$	0	0
	$M_{2}^{\left(2\right)}$	1	*i*	0	0	$-e^{i\frac{\phi_{m}}{2}}$	$ie^{i\frac{\phi_{m}}{2}}$	$-\sqrt{2}e^{i\frac{3\phi_{m}}{4}}$	$i\sqrt{2}e^{i\frac{\phi_{m}}{4}}$
*B*_3_	$M_{1}^{\left(3\right)}$	2	0	$\sqrt{2}e^{i\frac{\phi_{m}}{4}}$	$i\sqrt{2}e^{i\frac{3\phi_{m}}{4}}$	0	$2ie^{i\frac{\phi_{m}}{2}}$	$-\sqrt{2}e^{i\frac{3\phi_{m}}{4}}$	$i\sqrt{2}e^{i\frac{\phi_{m}}{4}}$
	$M_{2}^{\left(3\right)}$	0	2	$i\sqrt{2}e^{i\frac{\phi_{m}}{4}}$	$-\sqrt{2}e^{i\frac{3\phi_{m}}{4}}$	$2ie^{i\frac{\phi_{m}}{2}}$	0	$i\sqrt{2}e^{i\frac{3\phi_{m}}{4}}$	$\sqrt{2}e^{i\frac{\phi_{m}}{4}}$

In this table, pink cells indicate the regions with traveling waves while uncolored cells denote standing waves, and blue cells indicate the regions where the field vanishes. And *ϕ*_*m*_ = (2*m* + 1)*π*, where *m* is an integer.

Table 1 lists the field components associated with the degenerate eigenmodes of the structure shown in Figure 2 as expressed in the three different bases *B*_1,2,3_. The modes are expressed by the vector $M_{i}^{\left(j\right)}\equiv \left[a_{1},b_{1},a_{2},b_{2},a_{3},b_{3},a_{4},b_{4}\right]$ in each basis, where *i* represents the mode number and *j* denote the basis number. Note that there is a pure standing wave whenever the field components belonging to any domain have the same amplitude. On the other hand, if one of the field components vanishes, the wave is traveling. Evidently, in basis *B*_3_ the eigenmodes exhibit a hybrid-wave character with both standing and traveling waves coexisting as part of the same mode. Obviously, modes $M_{1,2}^{\left(1\right)}$ represent a standing wave that extends all over the structure. On the other hand, modes $M_{1,2}^{\left(2\right)}$ represent a standing wave that covers only part of the structure. Thus modes $M_{1,2}^{\left(3\right)}$ represent potential candidates for satisfying the conditions necessary for generating hybrid-wave modes. Indeed, this is confirmed by the field distribution of modes $M_{1,2}^{\left(3\right)}$ which exhibits the dual character of traveling and standing waves covering different domains of the resonators at the same time.

An important observation here is that for the perfectly closed resonator with no loss, the hybrid-wave modes occur only when the beam splitter is 50/50. A slight deviation from this condition removes the degeneracy and destroys the hybrid nature of the modes. This may seem to pose a challenge for experimentally observing these modes. However, realistic resonators are not perfectly closed but rather have losses due to optical absorption, radiation to free space, or due to the coupling to input and output channels. In turn, this will introduce an upper limit on the resonator’s quality factor and result in a finite bandwidth of operation, which relaxes the above constraint as discussed in detail in Supplementary Materials B and C.

**Figure 3:**
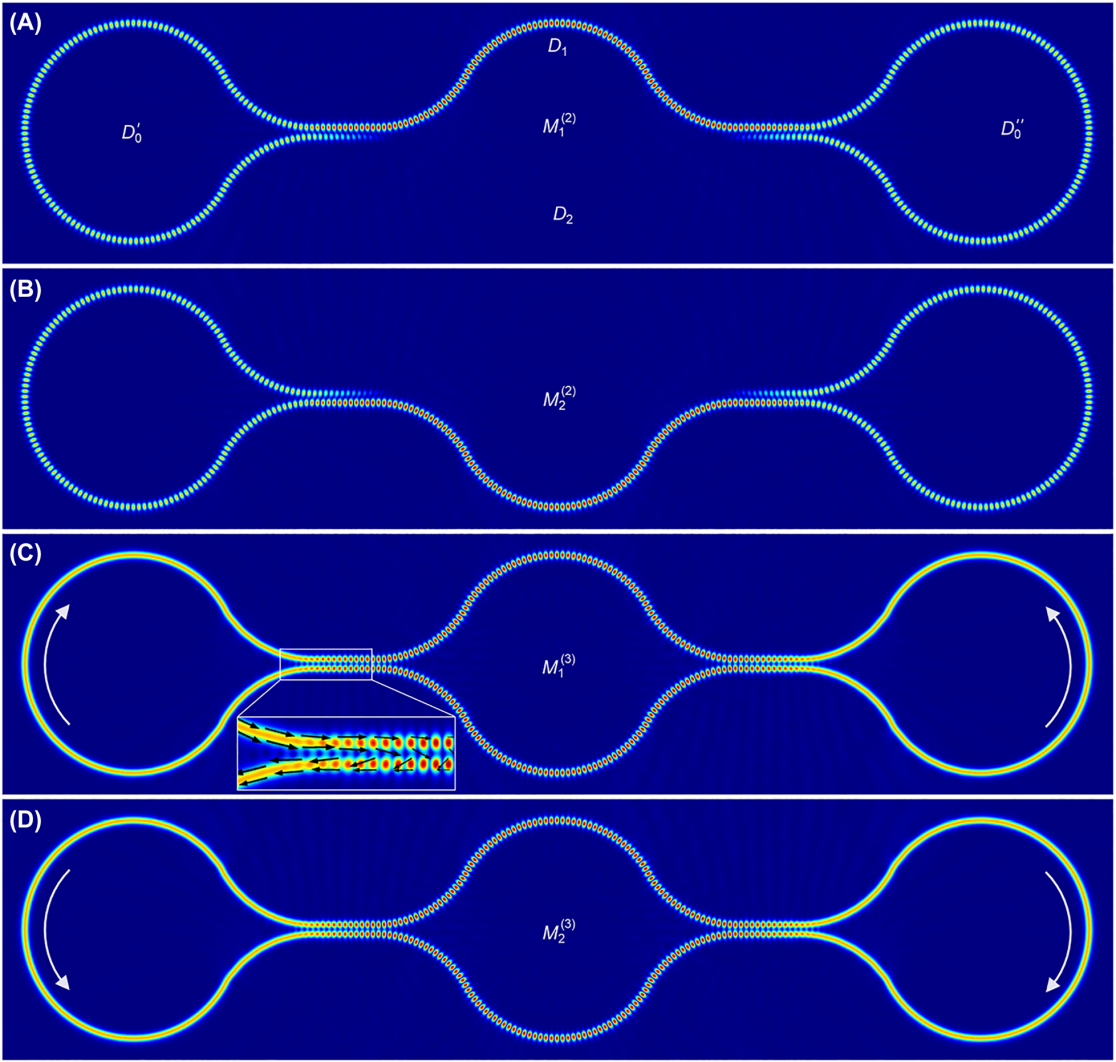
Eigenmodes of the hybrid-wave resonator presented in Figure 2. (A) and (B) are plots of electric field component perpendicular to the resonator’s plane (|*E*_*z*_|) associated with the two degenerate standing-wave modes $M_{1,2}^{\left(2\right)}$ which resides in domain *D*_0_ ∪ *D*_1_ and *D*_0_ ∪ *D*_2_, respectively, where $D_{0}=D_{0}^{\prime }\cup D_{0}^{\prime \prime }$ . These correspond to basis *B*_2_. On the other hand, (C) and (D) depict the field distribution corresponding to $M_{1}^{\left(3\right)}=M_{1}^{\left(2\right)}+M_{2}^{\left(2\right)}$ and $M_{2}^{\left(3\right)}=M_{1}^{\left(2\right)}-M_{2}^{\left(2\right)}$ , which feature a hybrid standing- and traveling-wave character. The white arrows in (C) and (D) indicate the traveling direction of traveling wave. The Poynting vectors (black arrows in inset of (C)) circulate around the loop in domain *D*_0_ and vanish in domain *D*_1,2_. The details of the geometry and material parameters used in these simulations are presented in Supplementary Materials C.

In order to verify the above predictions, we perform a finite element method (FEM) full-wave simulation (using COMSOL software package) of a realistic implementation for the structure shown in Figure 3. The details of the geometry and material parameters used in our simulations are presented in Supplementary Materials C. The eigenmodes generated by COMSOL package are in the basis *B*_1_ and are shown in Supplementary Materials C. Figure 3A and B shows the electric field distributions associated with the modes as represented in basis *B*_2_ which are generated via linear superposition of the degenerate modes $M_{1}^{\left(1\right)}$ and $M_{2}^{\left(1\right)}$ . On the other hand, the field distributions in basis *B*_3_ are depicted in Figure 3C and D. The nature of the waves can be deduced from the field distribution. Standing waves are visible through their interference pattern while traveling waves are characterized by uniform fields without interference. These plots are in agreement with the field distributions expected from Table 1 and indeed confirm the results obtained using the scattering matrix analysis above. The field distribution of modes $M_{1,2}^{\left(3\right)}$ deserves more attention. At the center of the middle sections (domains *D*_1,2_), the field features a standing wave while at the center of domains $D_{0}^{\prime }$ and $D_{0}^{\prime \prime }$ , they feature traveling waves. At the beam splitter junction, however, the field is neither a pure standing nor traveling wave. These regions represent transition domains where the wave gradually changes its character. From the Poynting vector point of view, this remarkable mode structure is enabled by the beam splitter junctions acting as interferometric mirrors for domains *D*_1,2_ while at the same time recirculating the power incident on them from domains *D*_0_ in a closed loop. Importantly, the standing-traveling wave nature observed in Figure 3C and D are characteristic of the eigenmodes of a single resonator structure and not associated with a particular steady state solution under certain engineered excitation [39]. Equally important is the fact that the traveling waves in the presented structure are part of the quasi-bound state within the resonator’s boundaries and not part of the leaked radiation waves outside the resonator as in the case of a finite Fabry–Perot or photonic crystal geometry for example. From a practical point of view, mapping these field distributions experimentally can be done only by using near-field probes, which is possible, but not an easy task. In Supplementary Materials E, we discuss a more practical scheme for accessing these modes by using input/output waveguides ports evanescently coupled to the various sections of the resonator.

## 4 Local perturbations and sensing applications

**Figure 4:**
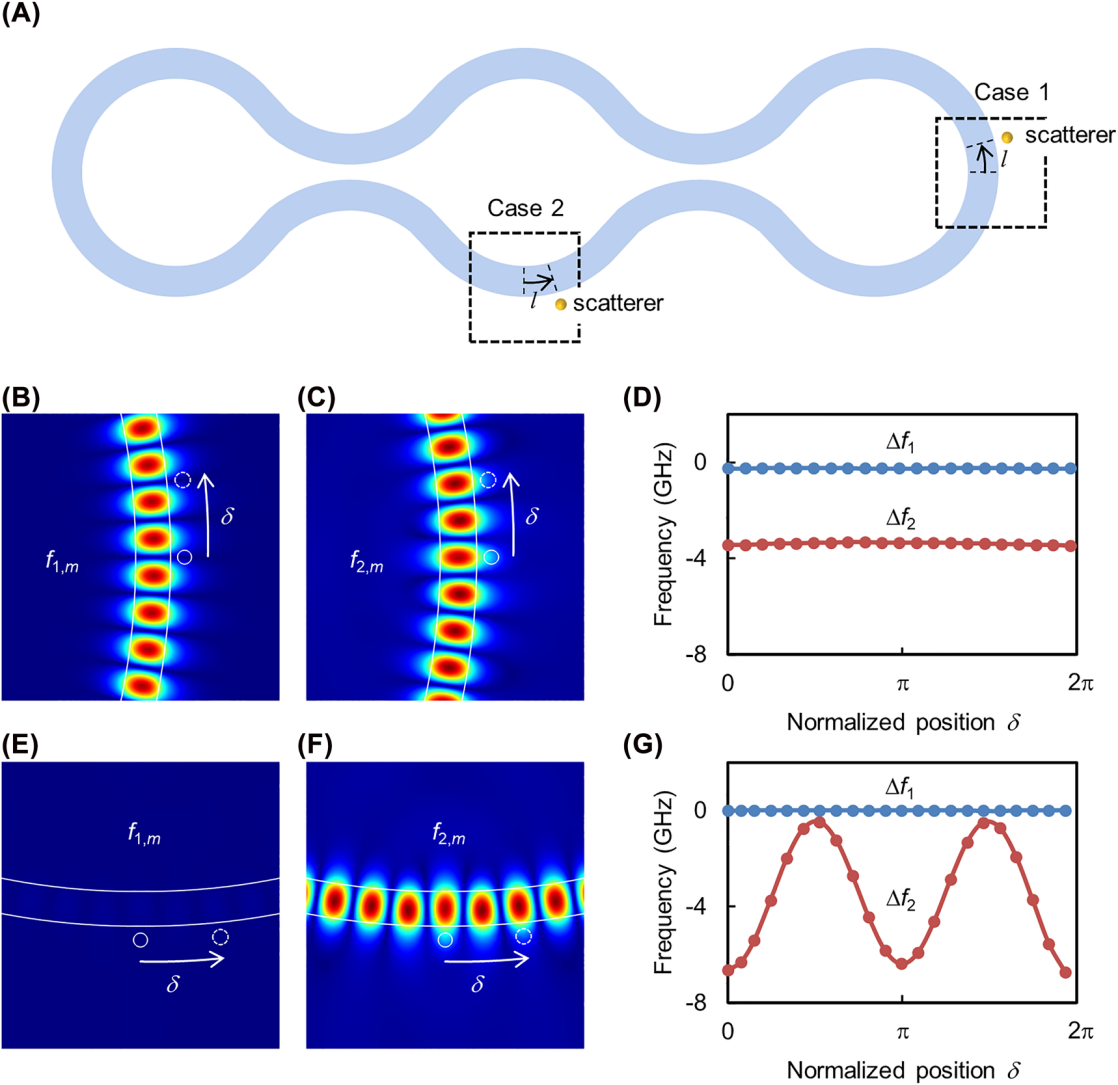
Effect of local perturbation. (A) A schematic of the resonator structure with nanoparticles scatterers added in the traveling wave (case 1) or standing waves (case 2) regions. In case 1, the presence of the scatterer generates two new optical modes that exhibit either a node (B) or an antinode (C) at the location of the particle with corresponding frequency shifts as shown in (D). In this case, the frequency shifts are independent of the particle location as long as it resides in the traveling wave domain. In case 2, the scatter leaves one of the modes intact with zero frequency shift (E) while at the same time introduces a perturbation to the second mode (F) with a frequency shift that varies with the location of the particle as expected (G). In both cases, *δ* = 2*π* corresponds to a distance of 0.5 μm along the perimeter. A rigorous derivation of these results as well as their intuitive explanations are discussed in the main text. In the figure, Δ*f*_*j*_ = *f*_*j*,*m*_ − *f*_*m*_ with *j* = 1, 2. The scatterers in panels (B), (C), (E) and (F) are indicated by small white circles.

In this section, we investigate the effect of local perturbation due to a small scatterer on the eigenmodes of the proposed hybrid-wave resonator—a problem relevant to sensing applications [9]. Given that the modes of the resonator shown in Figure 2A can be written in various bases, only one of which demonstrates the hybrid-wave character, one may wonder if this feature will have any consequences under more general conditions where the particular mode is not selectively excited. This section demonstrates that this is indeed the case. To illustrate this, we consider the situation where a scatterer (nanoparticle or a fiber tip for instance) is located within the evanescent field of the hybrid-wave resonator. In particular, we investigate the two scenarios where the scatterer is located either in the traveling- or standing-wave domains, as shown in Figure 4A.

Before we proceed, it is useful to review the situation for purely traveling-wave resonators (such as microring and microdisk arrangements) and purely standing-wave resonators (such as Bragg and photonic crystal arrangements). In the former, the scatterer breaks the rotational symmetry of the geometry and introduces coupling between the clockwise and counterclockwise modes, leading to a splitting of the eigenfrequency [40–42]. Importantly, this behavior is independent of the location of the scatterer. In the latter case, however, the situation is quite different. An optical mode that has an electric field node at the scatterer location along the resonator direction will not be affected by its presence. On the other hand, a mode that exhibits an antinode at the location of the scatterer will experience a shift in its eigenfrequency [43, 44].

To this end, we consider a small perturbation caused by a scatterer having a scattering matrix (Supplementary Materials F):

(3) $$S_{p}=e^{i\phi_{p}}\left[\begin{array}{cc} t & ir\\ ir & t \end{array}\right],$$

where *r* and *t* are reflection and transmission coefficients which are taken to be real numbers satisfying *r* ^2^ + *t* ^2^ = 1 (i.e. no loss); and *ϕ*_*p*_ = arcsin(*r*) is an overall additional phase.

### 4.1 Scatterer located along the traveling-wave domain

Here, we assume a scatterer located along the traveling-wave domain, say $D_{0}^{\prime \prime }$ , at a fixed distance from the resonator waveguide edge, as shown case 1 in Figure 4A. The angular position of the scatterer is defined by distance *l* (Figure 4A) and the corresponding phase shift *δ* ≡ 2*πn*_eff_ *lf*/*c*. By following the same approach used in deriving Eq. (1), we find that the resonant frequencies (see Supplementary Materials G):

(4) $$\left\{\begin{array}{c} \phi_{1,m}=\phi_{m},\;\\ \phi_{2,m}=\phi_{m}-2\phi_{p}.\; \end{array}\right.$$

where *ϕ*_*j*,*m*_ with *j* = 1, 2 indicates the new eigenfrequencies (due to the perturbation introduced by the scatterer) branched from the unperturbed eigenfrequency *ϕ*_*m*_. In other words, the perturbation shifts the frequency of only one mode while leaving that of the other unchanged. This can be explained by the fact that the new modes, arising because of the perturbation, both exhibit a standing-wave pattern, with the node of one mode and the antinode of the other located at the position of the scatterer. Moreover, Eq. (4) does not depend on the location of the scatterer along the perimeter of the resonator, as long as it lies in the traveling-wave domain. This behavior is exactly identical to the case of a scatterer introduced in the vicinity of traveling-wave resonator such as a microring or microdisk geometry [44, 45].

These predictions are confirmed by performing full-wave simulations, where the scattering was introduced via a nanoparticle. Figure 4B and C depict the field distribution of the perturbed modes around the particle. Note that, as expected, the particle modifies the field distribution and creates a standing-wave pattern. Moreover, the node of the first mode and the antinode of the second mode coincide with the particle location along the perimeter of the resonator, which is consistent with our theoretical predictions. As a result, the eigenfrequency of the first mode remains unchanged while that of the second mode experiences a constant shift that does not depend on the particle location, as shown in Figure 4D. As a side note, we remark that the blue dots representing the simulation data in Figure 4D do not exactly coincide with the zero axis as predicted by our scattering matrix analysis but rather exhibit a small shift. This can be explained by recalling that, in our analysis, we treat the particle as a Rayleigh scatterer, whereas in reality higher-order multipole terms must be considered in order to obtain more accurate results. To confirm this, we have performed additional numerical simulations for different particle sizes and indeed observed that this frequency shift decreases as the particle size is reduced (in fact we could not resolve the frequency shift for particles with radii less than 30 nm).

### 4.2 Scatterer located along the standing-wave domain

Next, we consider the case when the scatterer is located in the standing-wave region, for instance in domain *D*_2_, as shown by case 2 in Figure 4A. The resonant frequencies are given by (see Supplementary Materials G):

(5) $$\left\{\begin{array}{c} \phi_{1,m}=\phi_{m},\;\\ \phi_{2,m}=\phi_{m}-2\phi_{p}\left[1+{\left(-1\right)}^{m+1}\cos(2\delta )\right].\; \end{array}\right.$$

The mode corresponding to the eigenfrequency *ϕ*_1,*m*_ (Figure 4E) is associated with $M_{1}^{\left(2\right)}$ in Figure 3A, in which the electric field is zero at domain *D*_2_, leading to an unperturbed resonant frequency after adding the particle at domain *D*_2_. On the other hand, the eigenfrequency *ϕ*_2,*m*_ corresponds to a perturbation of mode $M_{2}^{\left(2\right)}$ in Figure 3A. Since mode $M_{2}^{\left(2\right)}$ is a pure standing wave at domain *D*_2_, the eigenfrequency *ϕ*_2,*m*_ varies with the angular position of the scatterer (Figure 4F and G). In Eq. (5), when *m* is an odd number, it is an antinode at the middle of *D*_2_ domain (*δ* = 0), *ϕ*_2,*m*_ experiences the maximum frequency shift −4*ϕ*_*p*_ from *ϕ*_*m*_; when *m* is an even number, it is a node at the middle of *D*_2_ domain, the scatterer will not alter the field much and the resonant frequency will stay the same, i.e. *ϕ*_2,*m*_ = *ϕ*_*m*_ at *δ* = 0. In both scenarios, *ϕ*_2,*m*_ will oscillate between *ϕ*_*m*_ an *ϕ*_*m*_ − 4*ϕ*_*p*_ as a function of *δ*. In our simulation, the fact that *m* is an odd number can be determined from Figure 3, and it is also verified by the electric field around the particle are shown in Figure 4F. The two eigenfrequencies varying with *δ* are shown in Figure 4G, consistent with Eq. (5).

From the above analysis, it is clear that a resonator exhibiting hybrid-wave modes will respond very differently to perturbations affecting the standing- or traveling-wave zones. In terms of applications, this can be useful in a number of ways. For instance, the larger splitting in the location of the field maxima in the standing-wave zone can be utilized for selective sensing by functionalizing [46] this exact location with receptors that can bind only to a particular molecule while at the same time use the traveling-wave zone for excitation and collection. On the other hand, one can instead use both zones for detecting the presence of more than one molecule (but only one at a time). This can be achieved by attaching different receptors to each zone and inferring the presence of a particular substance by measuring the degree of splitting. We plan to investigate these possibility in future works.

## 5 Conclusions

In conclusion, we have proposed a new concept for optical resonators that exhibit simultaneously co-existing standing and traveling waves as part of the field distribution of the same optical mode but occupying different locations along the resonator geometry. In addition, we have presented a specific example of a structure that implements this concept and verified its standing and traveling wave nature by using scattering matrix analysis and FEM full-wave simulations. We have investigated the robustness of the hybrid-wave feature and shown that the openness of the system allows for a larger bandwidth of operation and thus facilitates experimental observation. In addition, we have described a practical experiential scheme for probing the hybrid-wave nature by using several waveguide channels attached to various sections of the resonator geometry. Furthermore, we have discussed the implication of the hybrid-wave nature for sensing applications by investigating how the eigenmodes of such hybrid-wave resonator interact with a small scatterer located at different sections of the structure, demonstrating that the system’s response can be very different depending on the location of the scatterer along the standing- or traveling-wave sections. Another arena where hybrid-wave modes may prove useful is optical manipulation and trapping of particles. For instance, it is expected that a nanoparticle located in the traveling-wave zone will experience radiation pressure and lateral force acting in the direction towards the resonator, while a similar particle located in the standing-wave zone will in addition be subject to a trapping force along the perimeter of the resonator. Furthermore, actively tuning the beam-splitting values may allow for controlling the behavior of the resonator in real time and thus controlling its interaction with nanoparticles.

In addition, the existence of the hybrid-wave modes identified above, which to the best of our knowledge has not been known before, raises several fundamental questions in photonics, nonlinear and quantum optics applications. For instance, it is not *a priori* clear how such a resonator will behave under nonlinear conditions. Does it exhibit different nonlinear bistability responses than those observed in conventional ring resonators [47]? Does it provide any new features in terms of frequency comb generation? Can soliton crystals [48] form in the presence of hybrid-wave modes? Along similar lines, it is not clear to what extent the presence of the hybrid-wave modes will impact the dynamics and instability features of laser devices made of such resonators. In the quantum domain, it would be interesting to explore how quantum emitters located inside or in the vicinity of such resonators will behave. How would spontaneous emission and superradiance scale in different sections of the resonators? It has been shown previously that the interaction between atoms and electromagnetic waves featuring a standing field pattern depends on the type of resonator [29] (standing or traveling wave resonator). What makes these exploratory questions particularly interesting is that the proposed resonator exhibit transition regions (the beam splitter regions in Figure 2A that interpolates between the traveling- and standing-wave domains). Light–matter interaction in this region is expected to differ from its typical behavior in standard traveling- and standing-wave resonators, which may lead to interesting new effects. At the engineering level, our work also raises interesting questions. For instance, is there a fundamental size limit on building hybrid-wave resonators? Can one implement a small volume hybrid-wave mode? What would be the modes of these structures when implemented in material platforms that support plasmonic resonances? We plan to investigate these open questions as well as implementations in other platforms such as acoustics [49] and microwave [50] in future works.

## Supplementary Material

Supplementary Material Details
