# Supplementary material for: The missing link between standing- and traveling-wave resonators
Source: Nanophotonics. 2022 Aug 19;11(19):4427–37. doi: 10.1515/nanoph-2022-0304 (PMC11501156; doi:10.1515/nanoph-2022-0304)
Supplement: Supplementary file 1 — Supplementary Material Details [file j_nanoph-2022-0304_suppl_001.pdf]

The missing link between standing- and traveling-wave resonators
Supplementary Materials

Qi Zhong,[1,*] Haoqi Zhao,[2] Liang Feng,[3] Kurt Busch,[4,5]
Şahin K. Özdemir,[6,7] and Ramy El-Ganainy[1,8,†]

[1]*Department of Physics, Michigan Technological University, Houghton, Michigan 49931, USA*

[2]*Department of Electrical and Systems Engineering, University of Pennsylvania, Philadelphia, Pennsylvania 19104, USA*

[3]*Department of Materials Science and Engineering, University of Pennsylvania, Philadelphia, Pennsylvania 19104, USA*

[4]*Humboldt-Universität zu Berlin, Institut für Physik, AG Theoretische Optik & Photonik, D-12489 Berlin, Germany*

[5]*Max-Born-Institut, Max-Born-Straße 2A, 12489 Berlin, Germany*

[6]*Department of Engineering Science and Mechanics, The Pennsylvania State University, University Park, Pennsylvania 16802, USA*

[7]*Materials Research Institute, The Pennsylvania State University, University Park, Pennsylvania 16802, USA*

[8]*Department of Electrical and Computer Engineering, Michigan Technological University, Houghton, Michigan 49931, USA*

[*] qizhong@mtu.edu

[†] ganainy@mtu.edu

A. Implementation of hybrid-wave resonator using free-space optics

In the main text, we have discussed a possible implementation of hybrid-wave resonators using a integrated photonics platform. However, the concept of hybrid-wave resonators introduced in this work is general and can be implemented using other platforms. In this section, we demonstrate a possible realization based on free-space optics using the resonator geometry. As shown in Fig. S1, it consists of five mirrors and one 50/50 beam splitter. Depending on the actual physical design of the beam splitter, it could impart different phases on the transmitted and reflected waves as explained in Fig. S1. The method used in the main text can be also employed here to find the optical modes of the resonator in Fig. S1 for light with electric field polarized in a direction perpendicular to the plane of the resonator. Doing so, reveals the existence of two modes: the first of which, labeled as M_1, extends over domains $D_0 \cup D_1$ and has a resonant wavelength satisfying $2(l_1 + l_3 + l_4) \cdot \frac{2\pi}{\lambda} + 5\pi = 2m_1\pi$, where m_1 is an integer and the additional 5π term on the left side is due to the phase shift of the beam splitter (corresponding to the metal-coated beam splitter in Fig. S1) and four mirrors. The second mode, which we will refer to as M_2 occupies $D_0 \cup D_2$ and its resonant wavelength is given by $2(l_2+l_3+l_4)\cdot\frac{2\pi}{\lambda}+4\pi = 2m_2\pi$, where m_2 is integer and the additional 4π term is due to the phase shift of mirrors. By carefully adjusting the value of arm lengths l_1 and l_2, the

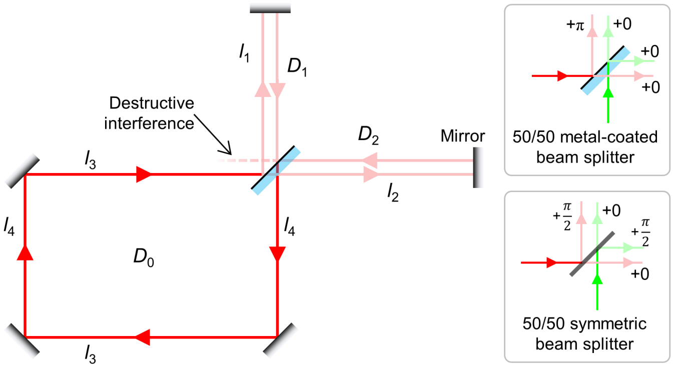

FIG. S1. A setup based on free-space optics that can support a hybrid-wave mode. As discussed in the text, the parameters of the resonators can be adjusted to produce destructive/constructive interference at the back output signals from the beam splitter in order ensure the formation of a standing-wave pattern in domains $D_{1,2}$ and a traveling-wave pattern in D_0. Two different kinds of beam splitters, i.e., metal-coated and symmetric beam splitters, are illustrated on the right.

resonance frequencies of these two modes can be tuned to form a degenerate pair. Following a similar argument to the discussion in the discussion in the main text, we thus see that a proper linear combination of mode M_1 and M_2 can form a traveling wave in domain D_0 and standing waves in domain D_1 and D_2. The light trajectory describing such a hybrid-wave mode is depicted in Fig. S1 by the red lines. Intuitively, the two oppositely traveling waves in $D_{1,2}$ form a standing-wave pattern while the constructive/destructive interference at the beam splitter back output (corresponding to the condition $2(l_2-l_1)\cdot\frac{2\pi}{\lambda}=[2(m_2-m_1)+1]\pi$) ensures the existence of only a traveling wave in the loop section of D_0 is satisfied. A similar conclusion also applies to the case of beams splitters with symmetric phase shifts such as that shown in Fig. S1 (for instance cube beam splitters or Pellicle beam splitters). Importantly, we note that the above discussion does not take into account free-space diffraction. Thus, it should be treated as a first-order approximation. More careful designs should be considered for actual free-space implementations.

B. Device bandwidth

As discussed in the main text, in the absence of any loss mechanism, the resonator structure shown in Fig. 2A will exhibit hybrid-wave modes only for 50/50 beam splitters. A carefully designed beam splitter may indeed exhibit a 50/50 splitting ratio at a chosen operating frequency, which we call ϕ_b (remember that $\phi \propto f$). This however will occur over a small bandwidth. In general, the resonant frequency of the resonator may not coincide with ϕ_b. This problem can be mitigated by tuning the length of the resonator throughout the design process to shift one of its resonant frequencies to ϕ_b. However, any deviation in the design parameters from their ideal target values will eventually break the degeneracy of the modes. This in turn puts stringent constraints on any realistic design and can hinder any efforts for experimental observation of such an effect. Fortunately, however, realistic optical resonators are not closed systems but rather experience loss due to radiation, material absorption and coupling to input/output channels. As we will show, this will relax the above constraint.

Let us consider the geometry in Fig. S2(a) where the resonator is evanescently coupled to a waveguide that is used for wave excitation and output signal collection. In this structure, the main sources of loss are coupling to the waveguide and radiation loss to free space

modes. This latter mechanism limits the resonator's quality factor even in the absence of a waveguide, as we discuss in detail in Supplementary Materials C. The finite element method (FEM) full-wave simulation shows that the beam splitting ratio is 50/50 only at one single frequency and can vary from 51/49 to 49/51 over five free spectral range (FSR) periods. As a result, the modes of the resonator are not degenerate as they would be desired but rather experience a splitting, as illustrated in Fig. S2(b). To further elucidate this behavior, we consider an input excitation from the bottom port of the waveguide and we compute the field coefficients a_2, b_2 and a_3, b_3 (see Fig. S2(a) for a full list of the field amplitudes and their respective locations along the resonator structure) by using the scattering matrix formalism:

$$\begin{aligned}
[a_{1-}, t_2]^T &= S_w[a_{1+}, p_1]^T, \\
[b_{1-}, t_1]^T &= S_w[b_{1+}, p_2]^T, \\
[a_{1+}, b_{1+}]^T &= S_c[a_4, b_2]^T, \\
[a_4, b_2]^T &= S_c[a_3, b_3]^T, \\
[a_3, b_3]^T &= S_c[a_2, b_4]^T, \\
[a_2, b_4]^T &= S_c[a_{1-}, b_{1-}]^T,
\end{aligned} \tag{B1}$$

where $S_w = \begin{bmatrix} \tau_w & i\kappa_w \\ i\kappa_w & \tau_w \end{bmatrix}$ is the scattering matrix between the waveguide and the resonator. Here, τ_w and κ_w are the field transmission and coupling coefficient, and they satisfy $\tau_w^2+\kappa_w^2 = 1$. The input amplitudes are taken to be $p_1 = 1$ and $p_2 = 0$. In the ideal scenario, $|\frac{a_2}{b_2}| = 1$ and $a_3 = 0$. The actual values of the various field components are plotted in Fig. S2(c) as a function of frequency for two values of the coupling coefficient: $\kappa_w = 0.2$ and $\kappa_w = 0.5$. As can be observed, in the former case, the standing-traveling wave feature persists only over a very narrow band centered around ϕ_b. In the latter case, however, the field values and ratios still represent standing and traveling waves in their corresponding resonator sections, at least to a good degree of approximation to the perfect behavior in a larger frequency band around ϕ_b. Evidently, the openness of the device thus facilitates the experimental observation of the hybrid standing and traveling wave features.

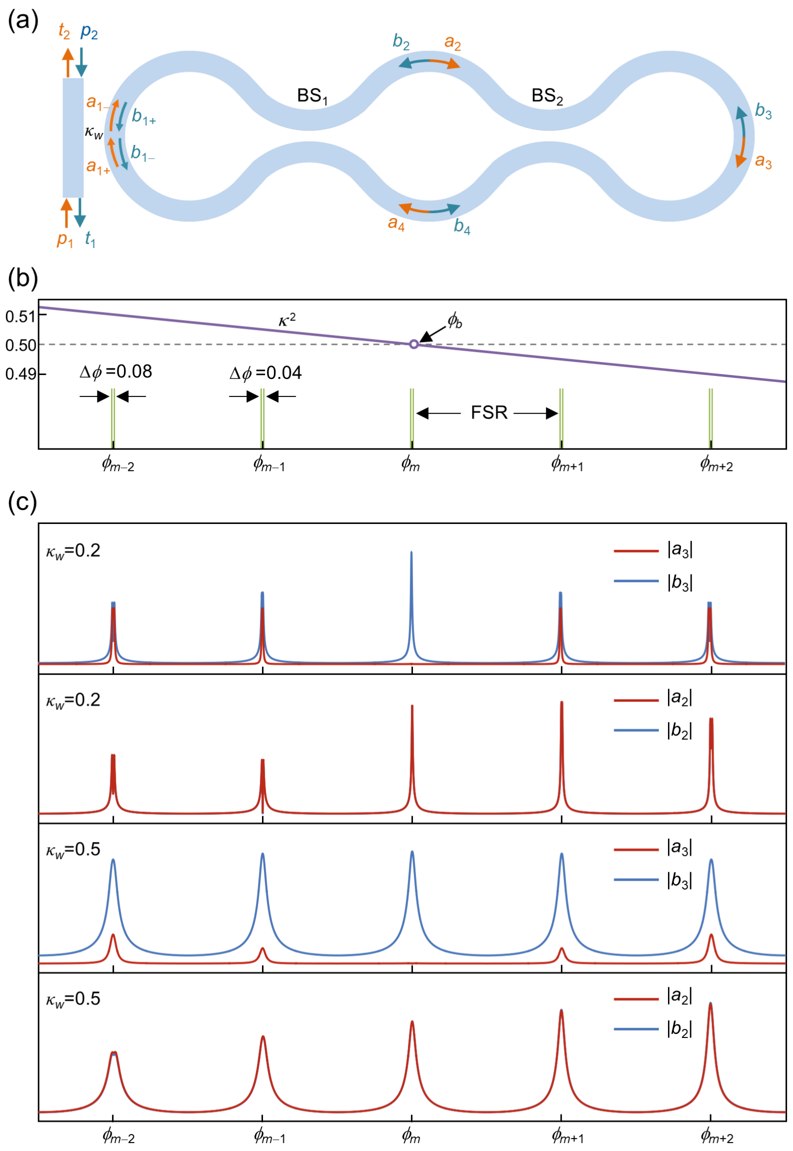

FIG. S2. The bandwidth of the device. (a) A schematic of a resonator structure similar to that of Fig. 2A with an additional evanescent coupled waveguide acting as an excitation and loss channel at the same time. (b) The dependency of the beam splitter coupling coefficient κ^2 as a function of frequency (obtained with FEM simulations) shows deviation from the ideal 50/50 operating point. Here ϕ_m coincides with ϕ_b and it is the ideal 50/50 operating point. However, the degeneracy of the modes centered around $\phi_{m\pm1}$ will be lifted with $\Delta\phi = 0.04$ due to the deviation of κ^2 from its ideal value of 0.5. (c) Plots of field amplitudes $|a_{2,3}|$ and $|b_{2,3}|$ as a function of frequency (calculated by using scattering matrix formalism) for two different values of the coupling to the waveguide, $\kappa_w = 0.2$ and 0.5, when the system is externally excited with $p_1 = 1$. As expected for a more lossy system, the hybrid-wave feature persists over a larger frequency range.

C. Simulation details

The simulation of eigenmodes in Fig. 3 in the main text is performed by using frequency-domain FEM available via COMSOL package. The geometry is shown in Fig. S3 and the parameters are listed in the figure caption. For fixed design parameters, the frequency (f_b) at which the beam splitting is 50/50, can be tuned by changing the beam splitter coupling length l_b. For example, in our simulations we take $l_b = 3$ μm in order to obtain $f_b = 192.1$THz (corresponding to a wavelength of 1.56 μm). In addition, a linear section of length l_0 is inserted into the top and lower sections to act as a design knob for controlling the location of the eigenfrequency of the resonator, ensuring that it coincides or at least overlaps with f_b. In our design, we take $l_0 = 0.08$ μm. For these parameters, FEM simulations indeed show that the two eigenmodes are almost degenerate with frequencies $f^+ = 192.11649$ THz and $f^- = 192.11647$ THz, as shown in Fig. S4. These two eigenmodes are $M_{1,2}^{(1)}$ in bases B_1. Due to the degenerate nature of these modes, any linear combination is also a mode. The field profiles plotted in Fig. 3 are constructed by using such appropriate superpositions as discussed in detail in the main text.

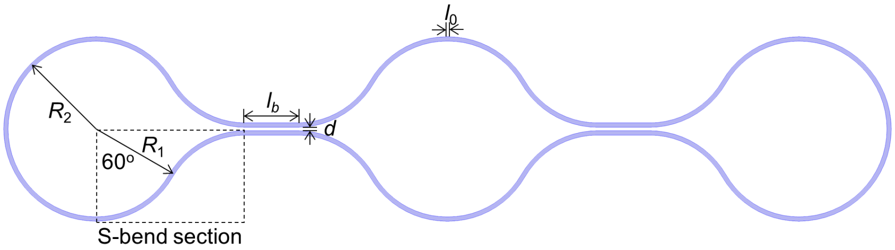

FIG. S3. The geometry of the resonator structure used in our FEM simulation. The waveguide of the resonator has a width of $w = 0.25$ μm, and a refractive index of 3.47, surrounded by a cladding with an index of 1.44 (typical values for silicon and silica at a telecom wavelength). The edge-to-edge gap in the directional coupler is $d = 0.2$ μm, and the length of the linear section in the coupling region is $l_b = 3$ μm. The S-bend section is constructed by connecting two 60° arcs with radius $R_1 = 5$ μm. The top and lower S-bend sections on each side of the resonator are then connected by a half-circle with $R_2 = R_1 + w/2 + d/2 = 5.225$ μm. A linear section with length $l_0 = 0.08$ μm is inserted into the top and lower middle sections in order to tune the eigenfrequency of the resonator equal to the frequency f_b at which the splitting ratio of the beam splitters is 50/50.

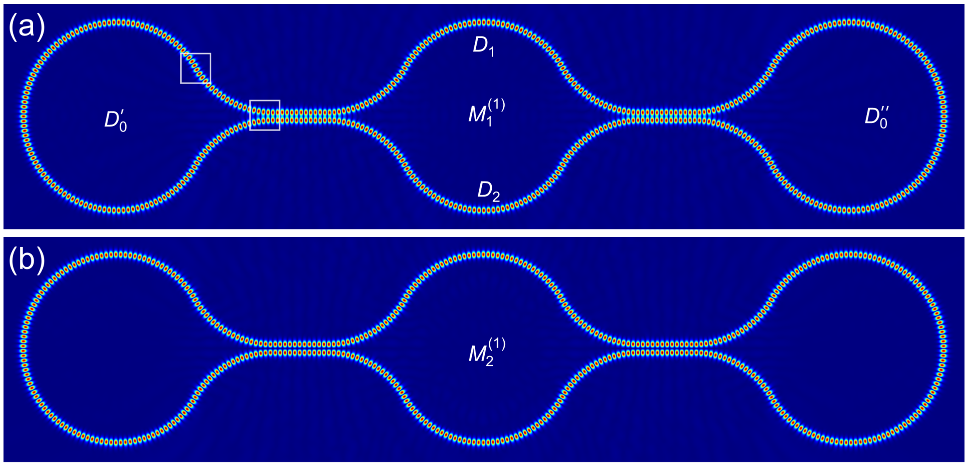

FIG. S4. COMSOL-generated electric field ($|E_z|$) distributions associated with the eigenmodes $M_{1,2}^{(1)}$ in bases B_1.

In order to evaluate the quality factor of the isolated resonator (i.e. without coupling to waveguides), we also extract the imaginary component of the resonant frequency from the FEM simulations. For the two degenerate modes in Fig. S4, the value of this imaginary component is ~ 1 GHz, which results in a quality factor of $Q \equiv \frac{f'}{2f''} \approx \frac{192 \text{ THz}}{2 \times 1 \text{ GHz}} = 10^5$. Further simulations indicate that the main source of the radiation loss in our structure are the S-bend sections and their connections to the beam splitter domains. For clarity, we illustrate two of these sections in Fig. S4(a). This is, however, not a fundamental source of loss and it is possible that further geometric optimization can reduce this loss and boost the quality factor.

D. An alternative design using integrated photonic platforms

The implementation presented in the main text is based on two loop mirrors. However, we note that this is not necessary. For instance, one can replace one of these loop mirrors with two ordinary mirrors as shown in Fig. S5(a). By adopting the same design parameters as those used in Fig. S3 and using full-wave simulations, we can compute the two degenerate modes of the systems. Figures S5 (b)–(g) present plots of these modes in different bases, where we clearly observe the hybrid traveling-standing wave nature in Fig. S5 (f) and (g). Here the mirror was introduced by using a 100 nm layer of silver.

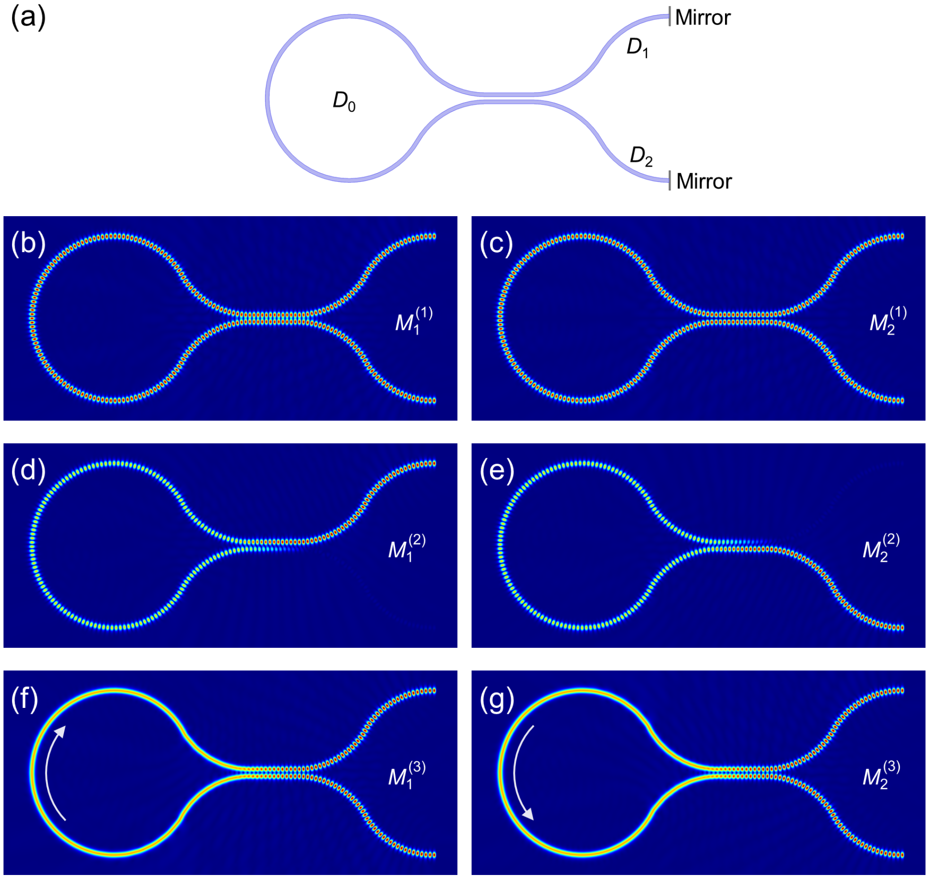

FIG. S5. A hybrid-wave resonator construed with mirrors. (a) The structure similar to that proposed in the main text after replacing the right most loop mirror with two conventional mirrors. The electric field distribution associated with the two degenerate eigenmodes (numerically obtained eigenfrequencies are $f^+ = 192.12172$ THz and $f^- = 192.12165$ THz) is plotted in different bases (b)–(g). The hybrid-wave nature is observed in the bases B_3, shown in (f) and (g).

E. Experimental implementation of observing hybrid-wave modes

In this section, we discuss how the hybrid-wave feature of the optical modes associated with the resonator geometry presented in the main text can be observed experimentally. First, owing to various possible representations of the modes due to degeneracy, one must ensure that the eigenmodes are excited in the correct bases, B_3 in our case. Second, one must verify the wave nature (standing or traveling) in each domain. In principle, this latter step can be performed by directly measuring the electric field distribution. However, this requires near-field measurements [1–3], which is not a trivial task. A modification of the above structure, however, can provide a means for probing the modal nature in a straightforward fashion by coupling each section to an external waveguide channel as shown in Fig. S6. The input and output ports of the waveguides are denoted by P_n with $n = 1, 2, ..., 8$. The power transmission coefficients from port P_m to P_n are labeled as T_{nm}.

Evidently, the eigenmode $M_1^{(3)}$ can be launched by exciting the resonator from ports P_1 or P_6 and setting all other inputs to zero. Similarly, mode $M_2^{(3)}$ can be excited via the ports P_2 or P_5. Here we focus on the eigenmode $M_1^{(3)}$ and we consider an excitation from P_1 only, keeping in mind that the same discussion applies to $M_2^{(3)}$. Due to the traveling-wave nature in domain D_0, it is expected to have $T_{11} = T_{61} = 0$. On the other hand, because the field distributions in domains $D_{1,2}$ correspond to a standing wave, we expect $T_{31} = T_{41} \neq 0$ and $T_{71} = T_{81} \neq 0$. This setup can thus provide direct information on the wave nature of the eigenmode in all domains without the need for any near-field measurements. These predictions are confirmed by performing FEM full-wave simulations using this modified structure.

Figure S6 also lists the power transmission coefficients T_{n1} at each output port due to an excitation from port P_1 as obtained by the FEM simulations as well as field distribution as obtained by FEM simulations where the traveling- and standing-wave patterns in domains D_0 and $D_{1,2}$ can be observed. Evidently, these results are consistent with the field distribution of eigenmode $M_1^{(3)}$. Note that T_{21} is much larger than T_{51} because output port P_2 is directly fed from input port P_1 as well as from the resonator, while port P_5 is fed only from the resonator. Finally, we would like to note that this strategy can also allow us to investigate the formation of exceptional surfaces [4, 5] in these hybrid-wave resonators which we plan to do in future works.

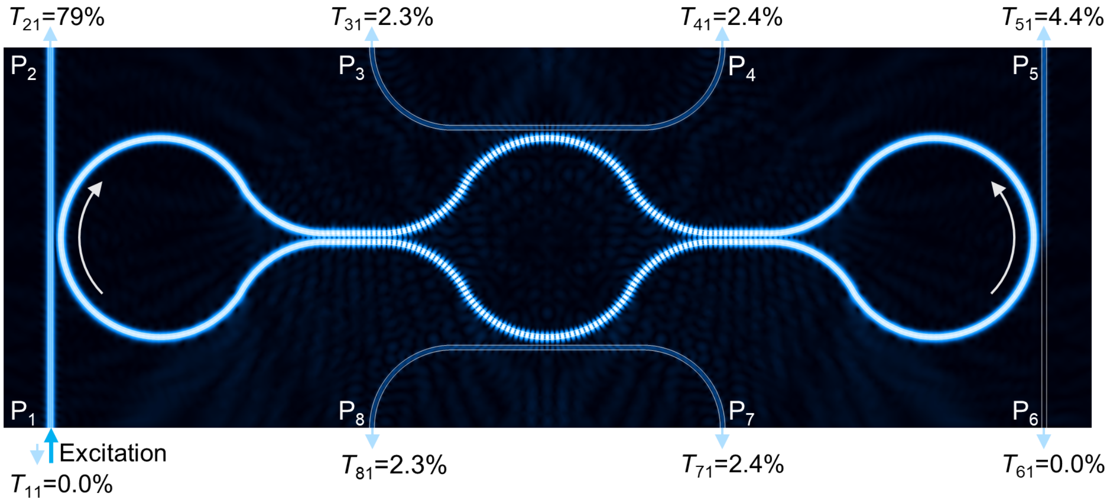

FIG. S6. The transmission of the resonator. A resonator structure similar to that of Fig. 3 with additional waveguide channels evanescently coupled at different sections. The standing or traveling wave nature of the modes can be probed by launching light into one of the waveguide ports (port P_1 in this particular case) and monitoring the output light from other ports. The values of the various power transmission coefficients are also listed on the figure. For example, the fact that $T_{31} \approx T_{41}$ indicates that the wave forms a standing-wave pattern in the central top section while $T_{61} = 0$ indicates that the wave component at the right side of the resonator is traveling. Similar arguments can be made about other domains. Color scale represents electric field component perpendicular to the resonator's plane ($|E_z|$).

F. Scattering matrix for a scatterer

Here we derive the scattering matrix S_p of Eq. (3) which is used to describe the scattering from a small scatterer. In the main text, we considered a nanoparticle as a scatterer but in the Rayleigh regime (i.e., the size of the particle is much smaller than the wavelength of the resonant light), all small scatterers behave in the same way regardless of their shape. Thus, to facilitate the analysis, here we will consider a small slab layer made of a different refractive index than the background index as a scatterer as shown in Fig. S7. It is straightforward to show that the field amplitudes shown in Fig. S7 are related by the transfer matrix equation [6]:

$$\begin{bmatrix} \tilde{a}_2 \\ \tilde{b}_2 \end{bmatrix} = \frac{1}{2n_1} \begin{bmatrix} n_1 + n_2 & n_1 - n_2 \\ n_1 - n_2 & n_1 + n_2 \end{bmatrix} \begin{bmatrix} e^{ikd} & 0 \\ 0 & e^{-ikd} \end{bmatrix} \frac{1}{2n_2} \begin{bmatrix} n_1 + n_2 & n_2 - n_1 \\ n_2 - n_1 & n_1 + n_2 \end{bmatrix} \begin{bmatrix} \tilde{a}_1 \\ \tilde{b}_1 \end{bmatrix}. \tag{F1}$$

Here, n_1 is the background refractive index and n_2 is the slab index, $k = 2\pi n_2/\lambda$ is the wave vector in slab and d is the slab thickness.

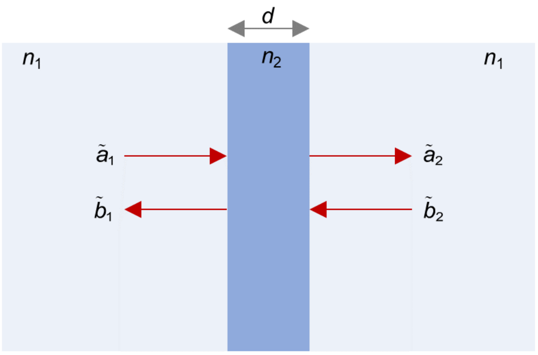

FIG. S7. A schematic diagram of the scatterer geometry used in our analysis in this section. It consists of a small slab made of refractive index n_2 embedded in a background of index n_1. In the limit of $n_2/n_1 \gg 1$ and $d \ll \lambda$, where λ is the light wavelength, we can derive the expression used for the scattering matrix in Eq. (F5). As shown in the main text, this form of the scattering matrix gives excellent agreement with results obtained using FEM simulations for nanoparticle scatterers. This can be understood by recalling that in the Rayleigh regime, all small scatterers basically behave identical regardless of the details of their shapes.

By substituting $\tilde{n} = n_2/n_1$ and $\theta = kd$, the total transfer matrix takes the form:

$$\mathcal{T} = \frac{1}{2\tilde{n}} \begin{bmatrix} 2\tilde{n}\cos\theta + i(\tilde{n}^2+1)\sin\theta & i(\tilde{n}^2-1)\sin\theta \\ -i(\tilde{n}^2-1)\sin\theta & 2\tilde{n}\cos\theta - i(\tilde{n}^2+1)\sin\theta \end{bmatrix}. \tag{F2}$$

We now consider the following limit for the scatterer parameters: $\tilde{n}^2 \gg 1$, $d \sim 0$ and $\theta \sim 0$. Under these conditions, $\mathcal{T}$ is given by:

$$\mathcal{T} = \begin{bmatrix} 1 + \frac{i}{2}\tilde{n}\theta & \frac{i}{2}\tilde{n}\theta \\ -\frac{i}{2}\tilde{n}\theta & 1 - \frac{i}{2}\tilde{n}\theta \end{bmatrix}. \tag{F3}$$

Importantly, in this limit, the matrix $\mathcal{T}$ still satisfies the power conservation. By using the relation between transfer and scattering matrices, we can finally obtain an expression for the scattering matrix S of the scatterer:

$$S = \frac{1}{1-ih} \begin{bmatrix} 1 & ih \\ ih & 1 \end{bmatrix}, \tag{F4}$$

where $h = \tilde{n}\theta/2$. Finally, by setting $r = \frac{h}{\sqrt{1+h^2}}$ and $t = \frac{1}{\sqrt{1+h^2}}$, we obtain

$$S = e^{i\phi} \begin{bmatrix} t & ir \\ ir & t \end{bmatrix}, \text{and } \phi = \arcsin(r). \tag{F5}$$

This is exactly the expression used in Eq. (3) and it gives consistent results with FEM simulations. Finally, we emphasize that this simple derivation presented here can be repeated for specific nanoparticle geometries but with more involved analysis. For details, see Refs. [7, 8].

G. Proof of particle scattering

In this section, we present a detailed derivation for Eq. (4) and Eq. (5) which describe the eigenfrequency splitting due to a perturbation by a small scatterer located at the traveling- and standing-wave sections of the resonator, respectively (see Fig. S8).

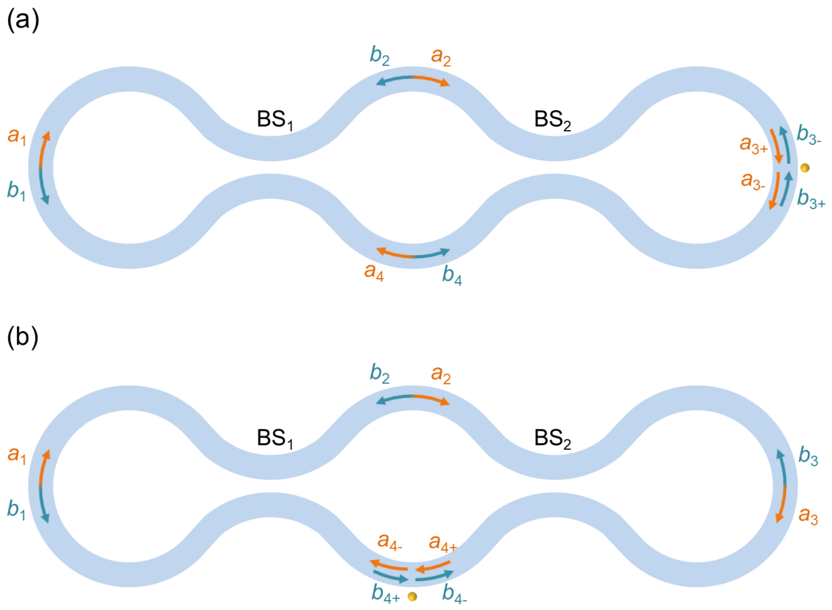

FIG. S8. A schematic of the resonator structure proposed in our work showing a nanoparticle serving as a perturbation. The particle can be located: (a) along the traveling-wave section; and (b) along the standing-wave section. Various field amplitudes used in our analysis are also depicted on the figure.

Scatterer located along the traveling-wave domain: Similar to the analysis of the resonant frequency in the main text, the relation between the field amplitudes in the presence of a scatterer located in the traveling-wave section (Fig. S8(a)) are given by:

$$\begin{aligned} [a_1, b_1]^T &= S_c[a_4, b_2]^T, \\ [a_4, b_2]^T &= S_c S_\delta^{-1}[a_{3-}, b_{3-}]^T, \\ [a_{3-}, b_{3-}]^T &= S_p[a_{3+}, b_{3+}]^T, \\ [a_{3+}, b_{3+}]^T &= S_\delta S_c[a_2, b_4]^T, \\ [a_2, b_4]^T &= S_c[a_1, b_1]^T. \end{aligned} \tag{G1}$$

Here, $S_\delta = \begin{bmatrix} e^{-i\delta} & 0 \\ 0 & e^{i\delta} \end{bmatrix}$ and δ characterize the angular position of the particle across the perimeter of te ring which is taken to be positive for the counterclockwise displacement. After some algebraic manipulations, we obtain:

$$[a_3^+, b_3^+]^T = S_{tr}[a_3^+, b_3^+]^T, \tag{G2}$$

where

$$S_{tr} = S_\delta S_c^4 S_\delta^{-1} S_p = -e^{i(\phi+\phi_p)} \begin{bmatrix} t & ir \\ ir & t \end{bmatrix}. \tag{G3}$$

The eigenvalues and eigenvectors associated with the matrix S_{tr} are given by $\lambda_1 = -\exp(i\phi)$, $\mathbf{u}_1 = [1, -1]^T$ and $\lambda_2 = -\exp[i(\phi + 2\phi_p)]$, $\mathbf{u}_2 = [1, 1]^T$. Finally by applying the consistency condition for obtaining the eigenfrequencies of the resonator, we find the solutions for ϕ:

$$\begin{cases} \phi_{1,m} = \phi_m, \\ \phi_{2,m} = \phi_m - 2\phi_p. \end{cases} \tag{G4}$$

As expected from our discussion in the main text, only one eigenmode is affected by the perturbation. Moreover, the frequency shift does not rely on the actual angular position of the particle (δ is absent from Eq. (G4)). These values for the new eigenfrequencies are consistent with the field distribution of the new modes. In particular, the field associated with mode $\phi_{1,m}$ corresponding to the eigenvector $\mathbf{u}_1 = [1, -1]^T = [a_3^+, b_3^+]^T$ has a node at the location of the particle, while that of mode $\phi_{2,m}$ with an eigenvector is $\mathbf{u}_2 = [1, 1]^T = [a_3^+, b_3^+]^T$ has an antinode at particle's position.

Scatterer located along the standing-wave domain: Next we consider the case depicted in Fig. S8(b) where the particle is located in the standing-wave section. In this case, the field amplitudes are related by:

$$\begin{aligned} [a_1, b_1]^T &= S_c[a_{4-}e^{i\delta}, b_2]^T, \\ [a_{4+}e^{-i\delta}, b_2]^T &= S_c[a_3, b_3]^T, \\ [a_3, b_3]^T &= S_c[a_2, b_{4-}e^{-i\delta}]^T, \\ [a_2, b_{4+}e^{-i\delta}]^T &= S_c[a_1, b_1]^T, \\ [a_{4-}, b_{4-}]^T &= S_p[a_{4+}, b_{4+}]^T. \end{aligned} \tag{G5}$$

Interestingly, Eq. (G5) does not lead to a simple consistency condition in the form $[a_1, b_1]^T = S_{st}[a_1, b_1]^T$. However, by eliminating $a_{1,3}$ and $b_{1,3}$, one obtains

$$\begin{aligned} [a_{4+}e^{-i\delta}, b_2]^T &= S_c^2[a_2, b_{4-}e^{-i\delta}]^T, \\ [a_2, b_{4+}e^{-i\delta}]^T &= S_c^2[a_{4-}e^{i\delta}, b_2]^T, \\ [a_{4-}, b_{4-}]^T &= S_p[a_{4+}, b_{4+}]^T. \end{aligned} \tag{G6}$$

By noting that $S_c^2 = i\exp(i\phi/2)\begin{bmatrix} 0 & 1 \\ 1 & 0 \end{bmatrix}$, we can finally obtain two independent consistency conditions:

$$\begin{aligned} \begin{bmatrix} a_2 \\ b_2 \end{bmatrix} &= ie^{i\frac{\phi}{2}} \begin{bmatrix} 0 & 1 \\ 1 & 0 \end{bmatrix} \begin{bmatrix} a_2 \\ b_2 \end{bmatrix}, \\ \begin{bmatrix} a_{4+} \\ b_{4+} \end{bmatrix} &= ie^{i\frac{\phi}{2}} \begin{bmatrix} 0 & e^{-i2\delta} \\ e^{i2\delta} & 0 \end{bmatrix} S_p \begin{bmatrix} a_{4+} \\ b_{4+} \end{bmatrix}. \end{aligned} \tag{G7}$$

It is worth commenting on these last relations. These are two independent conditions that describe independent sets of modes. This feature can be understood by referring to Fig. 3A and 3B where the modes are described in bases B_2. In this bases, the field of mode $M_1^{(2)}$ vanishes in the lower middle section while that of $M_2^{(2)}$ vanishes in the top middle section. As a result, a nanoparticle located as shown in Fig. S8 will affect only $M_2^{(2)}$ while at the same time leaving $M_2^{(1)}$ intact. This observation explains why two independent conditions for the eigenfrequencies arise in our analysis. By solving the above equation, we finally find the solutions for ϕ:

$$\begin{cases} \phi_{1,m} = \phi_m, \\ \phi_{2,m} = \phi_m - 2\phi_p[1 + (-1)^{m+1}\cos 2\delta], \end{cases} \tag{G8}$$

which completes the proof.

[1] H. E. Jackson, S. M. Lindsay, C. Poweleit, D. H. Naghski, G. N. De Brabander, and J. T. Boyd, Ultramicroscopy **61**, 295 (1995).

[2] M. Abashin, P. Tortora, I. Märki, U. Levy, W. Nakagawa, L. Vaccaro, H. P. Herzig, and Y. Fainman, Optical Express **14**, 1643 (2006).

[3] J. I. Ziegler, M. W. Pruessner, B. S. Simpkins, D. A. Kozak, D. Park, F. K. Fatemi, and T. H. Stievater, Nanophotonics **6**, 1141 (2017).

[4] Q. Zhong, J. Ren, M. Khajavikhan, D. N. Christodoulides, Ş. K. Özdemir, and R. El-Ganainy, Physical Review Letters **122**, 153902 (2019).

[5] S. Soleymani, Q. Zhong, M. Mokim, S. Rotter, R. El-Ganainy, and Ş. K. Özdemir, Nature Communications **13**, 599 (2022).

[6] B. E. A. Saleh and M. C. Teich, *Fundamentals of Photonics*, rhird ed. (Wiley, Hoboken, New Jersey, 2019).

[7] A. Venugopalan, D. Kumar, and R. Ghosh, Pramana **40**, 107 (1993).

[8] C. F. Bohren and D. R. Huffman, *Absorption and Scattering of Light by Small Particles* (Wiley, 1998).
